# Development and Validation of a Short Version of the Metric for the Observation of Decision-Making in Multidisciplinary Tumor Boards: MODe-Lite

**DOI:** 10.1245/s10434-021-09989-7

**Published:** 2021-05-11

**Authors:** B. W. Lamb, S. Miah, T. A. Skolarus, G. D. Stewart, J. S. A. Green, N. Sevdalis, T. Soukup

**Affiliations:** 1 Department of Urology, Cambridge University Hospitals NHS Foundation Trust, Cambridge, UK; 2 School of Allied Health, Anglia Ruskin University, Cambridge, UK; 3 Department of Urology, Buckinghamshire Healthcare NHS Trust, Amersham, UK; 4 VA Health Service Research and Development Center for Clinical Management Research, Ann Arbor, MI USA; 5 Dow Division of Health Services Research, Department of Urology, University of Michigan, Ann Arbor, MI USA; 6 Department of Surgery, University of Cambridge, Cambridge, UK; 7 Whipps Cross University Hospital, Barts Health NHS Trust, London, UK; 8 Center for Implementation Science, Health Service and Population Research Department, King’s College London, London, UK

## Abstract

**Background:**

Evidence-based tools are necessary for scientifically improving the way MTBs work. Such tools are available but can be difficult to use. This study aimed to develop a robust observational assessment tool for use on cancer multidisciplinary tumor boards (MTBs) by health care professionals in everyday practice.

**Methods:**

A retrospective cross-sectional observational study was conducted in the United Kingdom from September 2015 to July 2016. Three tumor boards from three teaching hospitals were recruited, with 44 members overall. Six weekly meetings involving 146 consecutive cases were video-recorded and scored using the validated MODe tool. Data were subjected to reliability and validity analysis in the current study to develop a shorter version of the MODe.

**Results:**

Phase 1, a reduction of the original items in the MODe, was achieved through two focus group meetings with expert assessors based on previous research. The 12 original items were reduced to 6 domains, receiving full agreement by the assessors. In phase 2, the six domains were subjected to item reliability, convergent validation, and internal consistency testing against the MODe-Lite global score, the MODe global score, and the items of the MODe. Significant positive correlations were evident across all domains (*p* < 0.01), indicating good reliability and validity. In phase 3, feasibility and high inter-assessor reliability were achieved by two clinical assessors. Six domains measuring clinical input, holistic input, clinical collaboration, pathology, radiology, and management plan were integrated into MODe-Lite.

**Conclusions:**

As an evidence-based tool for health care professionals in everyday practice, MODe-Lite gives cancer MTBs insight into the way they work and facilitates improvements in practice.

**Supplementary Information:**

The online version contains supplementary material available at 10.1245/s10434-021-09989-7.

Multidisciplinary tumor boards (MTBs) are the gold standard of cancer care delivery across the world.^1^ The accepted benefits of MTBs include improved adherence to best clinical practice, reduced geographic variability, timeliness of diagnostics and treatment, and improved outcomes including survival.^1^,^2^ Indirect benefits have been found including improved health care professional well-being, education, and quality assurance.^3^

The literature clearly shows that inefficiencies in MTB processes are commonplace.^1^,^3^ Factors such as professional hierarchies, lack of open discussion, failure to consider holistic information or patient views, and lack of personal knowledge of the patient all have an adverse impact on effective clinical decision-making.^4^ Improvement research has provided an evidence-based “tool kit” with which MTB members can identify factors that promote or hinder teams in reviewing patients holistically in a meeting and make recommendations that are both clinically sound and acceptable to patients.^5^,^6^

One such intervention is the MODe (Metric for the Observation of Decision-Making in cancer multidisciplinary tumor boards), which has been used to understand, assess, and improve MTB working Supplementary Fig. S1. 7–11 Development and validation of the tool have been reported previously.^4^,^7^–^9^ The MODe has been used to assess decision-making processes across different tumor types in different countries (Table 1).^7^–^19^

**Table 1:** Overview of literature using the Metric for Observation of Decision-Making (MODe)

Citation	Country	Tumor type	Use of MODe	Comments
Lamb et al.^7^	UK	Urologic cancers	Development and validation of MODe; 5 meetings (112 cases) Observed by surgeon and psychologist IRR: 112 cases, ICC 0.31-0.87	Scientific observational metrics can be reliably used by medical and non-medical observers in cancer MTBs to assess team decision-making.
Lamb et al.^8^	UK	Urologic cancers	MODe observational assessment Cross validation with a 29-question self-report Observation of 164 cases in 5 MTBs 47 surveys from MTB members (response rate 70 %)	The quality of teamworking and clinical decision-making in MTBs can reliably be assessed using observational and self-report metrics. MTB members have good insight into their own team performance.
Lamb et al.^10^	UK	Urologic cancers	MODe observational assessment Assessing effect of sequential MTB improvement interventions (e.g., MTBs checklist, MTB team training, and written guidance) Prospective longitudinal study: 16 months, 1421 patients	MODe can be used to evaluate the impact of QI interventions on MTB processes.
Jalil et al.^14^	UK	Urologic cancers, colorectal cancer, skin cancer, upper gastrointestinal cancer, head and neck cancer	MODe observational assessment Refinement of MODe Validation of use for assessment of video-recorded cases 683 multidisciplinary tumor board case -332 cases (9 urology MDMs) by 1 urologist -224 cases (6 urology boards) by 2 urologists -127 video-recorded case discussions (5 tumor types, over 8 MDMs) IRR: 224 cases, ICC >0.7	MODe scores correlate with decision efficacy. Video recordings offer a feasible, reliable method of assessing how MTBs work. MODe can be used across different tumor types Novice users can be trained to use MODe using video-recorded MTB meetings.
Shah et al.^15^	UK	Colorectal cancer	Modification of MODe to cMDT-MODe for use in colorectal cancer MDMs cMDT-MODe observational assessment 267 cases across 11 MDMs at single institution IRR: 76 cases, ICC 0.79 (0.70-0.92)	MODe can be adapted for use in specific tumor types, in this case to cMDT-MODe for colorectal patients.
Hahlweg et al.^16^	Germany	Dermatologic, gastrointestinal, gynecologic, head and neck, liver and biliary tract cancer, lymphoma and myeloma, neuro-oncologic, non-entity-specific oncologic, non-entity-specific surgical, thorax, and uro-oncologic cancer	MODe was adapted for use in German-speaking country MODe observational assessment 249 cases across 29 MTBs IRR: 39 cases, ICC.5 for all domains by end of study	MODe can be adapted for different languages and health care settings and provides reliable observational data.
Soukup et al.^11^	UK	Breast cancer	MODe observational assessment Assessing effect of co-designed intervention bundle (meeting breaks, change of room layout, meeting chair) MTB with 15 members, 1335 patient reviews	MODe can be used as part of “team audit and feedback” to improve teamwork in cancer care.
Lumenta et al.^18^	Austria	Mixed: not specified	MODe adapted to German language and culture as TB team performance assessment tool Clinical and nonclinical observers 244 patients in 27 MDMs IRR: cohorts of 11–141 cases, pairwise agreement 54–100 %	MODe was adapted to developed TB team performance tool in German-speaking country. Used to enabled the assessment of specialized multidisciplinary tumor boards with a special focus on teamwork patterns
Rosell et al.^19^	Sweden	Rare cancers: multidisciplinary tumor boards for penile cancer, anal cancer, and vulvar cancer	MODe and MOT observational assessment Electronic survey of health professionals from 6 MDMs 67 case discussions observed 125/241 (52 %) responses to survey IRR: 76 cases, agreement 0.86	MODe was used in a non-English-speaking health care setting. MODe can be used to assess video-conferenced MDT meetings.
Gandamihardja et al.^13^	UK	Breast cancer	MODe observational assessment 10 MDMs (346 patients). IRR: 116 cases, ICC 0.73-0.93	Breast cancer MTB evaluation via direct observation in a meeting is feasible and reliable.
Soukup et al.^12^	UK	Breast cancer, colorectal cancer, gynaecologic cancer	Observational assessment of team behaviors using 3 tools: MODe, Bales Interaction Process Analysis (Bales IPA), Measure of Case-Discussion Complexity (MeDiC). 3 MTBs with 44 members. 30 meetings filmed, 822 case discussions	MODe can be used together with other behavioral assessment metrics to unravel sociocognitive predictors of team DM quality. MODe used in conjunction with MeDiC can provide stratified assessment of performance accounting for case mix.
Scott et al.^17^	UK	Ovarian cancer	MODe adapted to gynaecologic oncology GO-MDT MODe GO-MODe observational assessment 223 MTB case discussions across 41 MDMs at 6 hospitals	MODe can be adapted for use in specific tumor types, in this case to GO-MODe for gynaecologic patients.
Soukup et al.^4^,^9^	UK	Breast cancer, colorectal cancer, urologic cancer	MODe Observational assessment combined with exploratory factor analysis and regression analyses to assess predictors of treatment decision Non-clinical and clinical observers 4 teams observed, 1045 case discussions IRR: 273 cases; ICC = 0.71–0.92	MODe can be used with other assessment tools to better understand the anatomy of MDT decsion making.

MTB, multidisclinary tumor board; IRR, ICC, interclass correlation coefficient; MDM, cMDT, GO-MDT

The MODe has been applied to show that the ability of an MTB to reach a clinical decision is associated with high-quality comprehensive and necessary information (from case history, radiology, pathology) available at the point of decision-making, team contribution, and the order of cases in a meeting.^11^,^12^ The MODe has been used to provide objective validation of the impact that quality improvement interventions such as meeting preparation, team training, meeting breaks, improved chairing style, and room layout has on outcomes including meeting time, ability to reach management recommendations, and maintenance of decision-making quality during long meetings.^12^ Moreover, using factor analysis, the MODe has demonstrated that a complete patient profile and representation by all core disciplines are necessary to maximize the ability of an MTB to reach management recommendations for all cases.^9^

Users of the MODe (Table 1) have provided a useful critique of the tool applied to a range of clinical and research settings. During real-life MTB meetings, it can be difficult for observers to differentiate individual variables when scoring.^13^,^16^ Moreover, previous content validation of the MODe has suggested that fewer factors are desirable.^9^ This sentiment has been echoed by some of the health care professionals we have trained to use the MODe in clinical practice, who expressed a desire for a simpler tool that can be used for clinical audit (unpublished data).

The current study aimed to produce a tool that would retain and simplify the most important elements of the MODe,^7^ and that could be used by health care staff, who are ultimately the end users of MTB processes. We therefore developed the MODE-Lite, a more user-friendly version of the MODe, which is intended for use in clinical practice to assist teams in quality improvement and streamlining of processes. Specifically, the objectives of this study were (1) to retain validity by involving experts in tumor board improvement in the construction of the tool, building on previous content validation of the MODe^9^ (phase 1), (2) to validate the MODe-Lite externally against a validated measure of case complexity (MeDiC)^20^,^21^ in line with previous research^12^ (phase 2), and (3) to ensure feasibility and reliability by assessing the use of the MODe-Lite via several teams of expert and novice users across different tumor types (phase 3).

## Methods

### Study Design

This study was a retrospective cross-sectional observational psychometric investigation.

### Study Setting

The study took place across three university hospitals in metropolitan areas of the United Kingdom between September 2015 and July 2016. Availability sampling was used to identify MTBs from the UK National Health Service (NHS) that represent the most common cancer types. Three MTBs were identified and participated in the study including breast, colorectal, and gynecologic MTBs.

### Participants

The study participants were 44 tumor board members: 15 breast, 15 colorectal, and 14 gynecologic professionals. The tumor boards had the same composition of surgeons (*n* = 12), oncologists (*n* = 6), cancer nurse specialists (CNS) (*n* = 12), radiologists (*n* = 6), histopathologists (*n* = 5), and coordinators (administrative role, *n* = 3). The groups were at the attending level, with an average 9 years of experience (minimum, 2 years; maximum, 22 years). A detailed team composition breakdown has been published previously.^12^,^22^,^23^ Ethical approvals were given by the North West London Research Ethics Committee and locally by the participating hospitals as part of the original research.^23^ Oral and written consents were given by the team members.

All case discussions during the study period were video-recorded, including discussions on suspected or confirmed cancer. The study included 146 consecutive case discussions from six tumor boards. The dataset is available on Zenodo.^24^ The sample size was determined using G*Power 3^25^ for a priori power analysis with a two-tailed test, an effect size of 0.6, a probability of 0.05, and a power of 0.90.

### Tool Development and Validation Phases

We developed MODe-Lite from the original MODe instrument^7^ and its previous content validation^9^ positing a reduction in the number of the original items. A multi-phased approach was undertaken in developing MODe-Lite.

In phase 1 (objective A), two meetings were held with experts (B.W.L., a clinical researcher and consultant surgeon, and T.S., a clinical researcher and psychologist) with more than 5 years of experience in the use of MODe and evidence^4^,^9^,^12^ of proficiency with inter-assessor reliability higher than 0.70.^26^ The aim was to design a new tool and to assess its content validity against MODe’s previous content validation.^9^

In phase 2 (objective B), assessor 1 (B.W.L.) scored a sample of 146 cases that had previously been assessed with MODe using the revised shorter version of the tool (MODe-Lite). The aim was to assess convergent validity with the original tool.^7^ We hypothesized (H1) that the MODe-Lite domains would correlate positively with the relevant items of the original MODe,^7^ in line with the factor model proposed in previous research.^9^ In addition, we aimed to validate MODe-Lite externally against case complexity as measured by the previously validated Measure of Case-Discussion Complexity (MeDiC).^20^,^21^ We hypothesised (H2) that MODe-Lite would positively correlate with the MeDiC tool, in line with the previous research using the MODe instrument.^12^

In phase 3 (objective C), both assessors (B.W.L. and T.S.) trained a new assessor (S.M., a consultant surgeon) in the use of the shorter version (MODe-Lite) on video-recorded tumor boards during two 2-h-long sessions. The assessors (B.W.L. and S.M.), blinded to the each other’s scores, scored a subset of 60 cases. Disagreements were subsequently discussed during a single 2-h data-review session to understand how the scoring of the shorter tool could be improved. The aim was to determine feasibility and inter-assessor reliability in the use of MODe-Lite.

### Statistical Analysis

The validity of MODe-Lite was assessed using a widely used measure, the item-content validity index (I-CVI).^27^,^28^ The criteria for item acceptability depends on the number of experts rating the items.^27^,^28^ If the experts are fewer than five, all five must agree for the item to be retained.^27^,^28^

We performed convergent validity analysis of MODe-Lite by assessing the correlation between individual domains and the original MODe instrument (the individual items of MODe and the global score). We also used the overall MODe-Lite score for the item-total correlation.

We assessed the reliability between the two assessors (B.W.L. and S.M.) using kappa coefficients for categorical items (i.e., the individual items of MODe-Lite) and interclass correlation coefficients (ICCs) for continuous items (i.e., the global scores). For the ICCs, a generally accepted reliability coefficient of 0.70 or higher was used,^26^ whereas for the kappa coefficients, the following criteria applied: fair agreement (0.21–0.40), moderate agreement (0.41–0.60), substantial agreement (0.61–0.80), and almost perfect agreement (0.81–1.00).^29^ Cronbach's alpha was calculated to assess the internal consistency for each MODe-Lite domain (i.e., how closely related these domains are against their corresponding items in the original MODe tool).

## Results

### Descriptive Analysis

Table 2 shows summary statistics for the individual domains of MODe-Lite across the entire dataset (*n* = 146) comprising three tumor boards, namely, breast, colorectal and gynecologic boards. The holistic input and clinical collaboration domains scored lowest, indicating overall lower quality. Pathology and clinical input scored highest, indicating better quality.

**Table 2:** Summary statistics for the MODe-Lite domains^a^

MODe-lite domain	M	SD	Mdn	IQR	Min	Max
Clinical input	2.25	0.68	2	1	1	3
Holistic input	1.25	0.52	1	0	1	3
Clinical collaboration	1.76	0.76	2	1	1	3
Pathology	2.34	0.84	3	1	1	3
Radiology	2.15	0.96	3	2	1	3
Management plan	2.20	0.74	2	1	1	3
Global score	11.95	2.50	12	4	6	18

MODe, Metric for the Observation of Decision-making; MODe-Lite, user-friendly version of the MODe; M, mean; SD, standard deviation; Mdn, median; IQR, interquartile range; Min, minimum; Max, maximum

^a^Score range for the individual domains is 1 to 3, and for the global score it is 6 to 18. Higher scores indicate better quality. Note. Total (*n* = 146), breast (*n* = 40), colorectal (*n* = 31), gynecologic (*n* = 75)

### Phase 1: Item Content Validation (Objective A)

Two focus group meetings were held between two expert assessors (B.W.L. and T.S.) for content validation using all 12 items from the original MODe instrument.^7^ Guided by the previous content validation of the MODe tool with a large sample (n = 1045) using exploratory factor analysis,^9^ the factor model containing clinical and holistic (patient history; oncologists’, surgeons’, and nurses’ inputs; psychosocial information; comorbidities; patient view), radiology (radiology information and radiologists’ inputs), and pathology (pathology information and pathologists’ input) components received full agreement (I-CVI = 1) for inclusion into MODe-Lite.

**Fig. 1.**
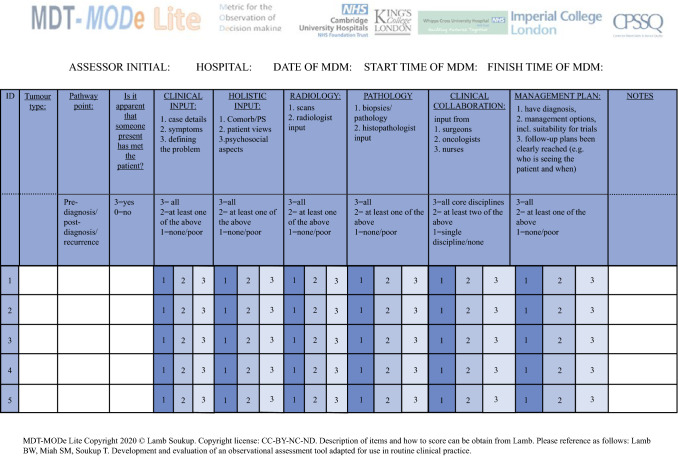
Copy of the MODe-LITE tool. MODe-LITE Copyright 2021 © Soukup Lamb under CC-BY-NC-ND

Because of the substantial research evidence supporting holistic information^1^,^4^,^5^,^9^,^11^,^30^,^31^ and clinical collaboration^1^,^4^,^5^,^11^,^32^–^34^ adequately captured in the assessments for team quality improvements,^10^,^11^ it was agreed by the expert assessors (I-CVI = 1) that they should be scored separately, resulting in three domains: clinical input, holistic input, and clinical collaboration. Pathology and radiology were retained as separate domains (I-CVI = 1), in line with the factor analysis.^9^ Management plan, an outcome variable in the factor analysis and previous research,^9^ also was retained (I-CVI = 1) as a separate item. Therefore, a total of six domains received full agreement for inclusion in MODe-Lite by the expert assessors (I-CVI = 1) and were subjected to further validity and reliability testing. The tool is represented graphically in Fig. 1.

### Phase 2: Item Convergent Validity, Reliability, and External Validity (Objective B)

The six domains of MODe-Lite were next validated against the global scores of MODe-Lite, the original MODe,^7^ and the MeDiC tool,^20^,^21^ (in line with previous research)^12^, respectively. All the MODe-Lite domains showed significant positive correlation with the MODe-Lite global score (Table 3), indicating good convergent validity across all six domains. In addition, significant positive correlation was evident against the global score for the original MODe tool, indicating good external validity and support for the H1 (i.e. MODe-Lite will correlate positively with the relevant items of the original MODe). The exception was pathology, for which the correlation coefficient, although positive, did not reach statistical significance. Significant positive correlation also was evident against the global score for the MeDiC tool, further indicating good external validity and support for the H2 (i.e. MODe-Lite will correlate positively with the MeDiC tool in line with previous research^12^).

**Table 3:** Item convergent validity, reliability, and external validity for the MODe-Lite

MODe-lite domain	*n*	MODe-Lite global score		MODe global score		MeDiC global score	
		*R*	*P* value	*R*	*P* Value	*R*	*P* Value
Clinical input	146	0.60	0.001	0.41	0.001	0.12	0.159
Holistic input	146	0.58	0.001	0.43	0.001	0.39	0.001
Clinical collaboration	146	0.70	0.001	0.51	0.001	0.38	0.001
Pathology	146	0.25	0.002	0.12	0.149	0.03	0.740
Radiology	146	0.54	0.001	0.52	0.001	0.33	0.001
Management plan	146	0.77	0.001	0.44	0.001	0.25	0.003
MODe-LITE global score	146	–	–	0.71	0.001	0.41	0.001

MODe, Metric for the Observation of Decision-making; MODe-Lite, user-friendly version of the MODe; MeDiC, Measure of Case-Discussion Complexity; *n,* sample size; *r,* Pearson’s correlation; *P,* statistical significance value (*P* < 0.05)

Further external validation was performed against the corresponding MODe items in line with the content validation in phase 1 and previous research.^9^ Table 4 shows significant positive correlations throughout between the 6 MODe-Lite domains and their corresponding 12 MODe items. This finding provided further support for H1.

**Table 4:** External validity and internal consistency for MODe-Lite against the original MODe tool^a^

		MODe item						
MODe-LITE domain	*n*	*r*	*P* value	*r*	*P* value	*r*	*P* value	Cronbach’s alpha
		Patient history						
Clinical input	146	0.52	0.001					0.64
		Psychosocial information		Comorbidity information		Patient views		
Holistic input	146	0.36	0.001	0.46	0.001	0.28	0.001	0.66
		Surgeon input		Oncologist input		Nurse input		
Clinical collaboration	146	0.21	0.012	0.48	0.001	0.45	0.001	0.58
		Radiologist input		Radiologist information				
Radiology	146	0.53	0.001	0.76	0.001			0.80
		Pathologist input		Pathology information				
Pathology	146	0.76	0.001	0.78	0.001			0.90
		Decision reached						
Management plan	146	0.56	0.001					0.71

MODe, Metric for the Observation of Decision-making; MODe-Lite, user-friendly version of the MODe; *n,* sample size; *r,* Pearson’s correlation; *P,* statistical significance value (*P* < 0.05)

^a^A traffic-light system for a visual guide was used to indicate how well each set of MODe items relates to its corresponding MODe-Lite domain: green represents good internal consistency, and amber represents fair internal consistency.

The Cronbach alpha, measuring how closely related each set of MODe items is as a domain of MODe-Lite, was good for pathology, radiology, and management plan, and somewhat weaker for the clinical, holistic inputs, and clinical collaboration, warranting further testing on a larger sample.

### Phase 3: Inter-Assessor Reliability and Feasibility in the Use of the Tool (Objective C)

Inter-assessor agreement on MODe-Lite was examined using kappa coefficients for categorical (i.e., the individual items of MODe-Lite) and ICCs for continuous (i.e., the global scores) variables on a subsample of 60 cases (40 % of the total). Good reliability between the two raters (B.W.L. and S.M.) was evident across all six domains, with the ICCs for global score higher than the generally accepted 0.70^26^ and the kappa coefficients for individual items ranging from moderate to almost perfect agreement. The tool was reported feasible and straightforward to use by both assessors.

A post-scoring data-review session was undertaken to understand how the scoring of the tool could be improved in the two domains that initially appeared to have lower ICCs: holistic input (0.64) and management plan (0.68). Disagreement with regard to scoring of holistic information was perceived to be a limitation of data quality, specifically, sound quality. Scoring of information on holistic aspects of care in MODe-Lite was not anchored to specific members of the MTB, nor to specific terminology, and therefore was easier for observers to miss when the sound quality of the video recordings was poor.

Another limitation of data quality was disagreement on scoring of the management plan. The observers noted that the MTBs used abbreviations for follow up plans (e.g., abbreviation for a follow-up pathway) that were specific to that team or organization. However, abbreviations or jargon relating to the disease or investigations are more universally understood (e.g., CT scan). Locally specific abbreviations might not be understood by external assessors.

After review and discussion of specific cases of disagreement, the majority were settled in agreement, and revised scores for ICC were recorded (Table 5). A minority of cases remained in disagreement between the observers, which related to perceived differences in the application of the scoring, particularly for management plan. Specifically, the word “treatment” was thought to apply only when treatment was recommended. Where patients were found not to require treatment, it was thought that the word “management” was more appropriate. Otherwise, despite the formation of a good management plan, a top score could not be awarded. The assessors agreed on a slight modification of the anchor behavior for management plan, with a change in wording from “treatment options,” to “management options.”

**Table 5:** Inter-assessor reliability coefficients for the MODe-Lite domains

MODe-Lite domain	*n*	Assessor 1		Assessor 2		Kappa
		M	SD	M	SD	
Clinical input	60	1.98	0.73	1.95	0.77	0.64
Holistic input	60	1.20	0.48	1.25	0.54	0.89
Clinical collaboration	60	1.67	0.75	1.53	0.62	0.60
Radiology	60	2.00	0.97	1.95	0.99	0.85
Pathology	60	2.63	0.66	2.70	0.65	0.70
Management plan	60	2.03	0.74	1.82	0.77	0.97
Global score	60	11.52	2.50	11.20	2.45	0.84^a^

MODe, Metric for the Observation of Decision-making; MODe-Lite, user-friendly version of the MODe; *n,* subsample size; M, mean; SD, standard deviation

^a^Intraclass correlation coefficient (ICC) values. Kappa coefficients can be interpreted as follows: 0.21–0.40 (fair agreement), moderate agreement (0.41–0.60), substantial agreement (0.61–0.80), almost perfect agreement (0.81–1.00).

The change from scoring a Likert scale of 5 points to 3 points was thought by the observers to make scoring easier. The anchor behaviors of MODe-Lite were of a more categorical nature (with scores assigned for the accumulation of different types of behaviors) than those of MDT-MODe,^7^ which required users to quantify a given amount of a composite behavior (e.g., contribution of a particular speciality). The assessors found that it was easier, and therefore quicker, to apply a score to MODe-Lite than to the original MODe. This increased ease of scoring was thought by the observers to be important when several domains were scored in real time and made the task more feasible.

## Discussion

The current study aimed to improve the utility of an existing observational assessment tool (MDT-MODe)^7^ by designing and validating a shorter version of MODe (i.e., MODe-Lite) for use in routine clinical practice. We demonstrated good item-content validity, with the convergent validity of our H1 supported. The MODe-Lite domains correlated positively with the relevant items of the original MODe,^7^ in line with the factor model proposed in previous research (objective A).^9^ In addition, H2 was supported as we were able to demonstrate external validity against the MeDiC tool^20^,^21^ (objective B) in line with previous research that used MODe.^12^ We also demonstrated good inter-assessor reliability and feasibility in the use of the tool (objective C). Internal consistency was good across all MODe-Lite domains, but clinical input, holistic input, and clinical collaboration showed weaker consistency, warranting further testing with a larger sample.

Although clinical input, holistic input, and clinical collaboration had loaded onto a single factor in previous research, suggesting that they should be grouped into a single domain for scoring,^9^ based on the evidence and the needs of the tumor boards, their separation is critical for adequate team assessment and provision of feedback for quality improvements.^10^,^11^ For instance, evidence shows that holistic inputs tend to be underrepresented in the decision-making of tumor boards, yet are essential for their ability to reach^4^,^9^ and subsequently implement a treatment recommendation,^35^,^36^ and are encouraged by the relevant guidelines.^31^,^37^ Therefore, scoring of the holistic input separately and pulling it apart from the other three items can help in assessing how well a team covers this aspect and how to improve it. Similarly, evidence shows that clinical collaboration of tumor boards can be suboptimal. However, it is critical for effective decision-making,^4^,^9^,^35^,^36^ and thus, it is important that this item be scored separately as well.

## Study Implications

The MODe tool^7^ has been used in studies across many tumor types and applied to different languages in different countries (see Table 1). Its broader impact on the literature surrounding MTB transformation has been demonstrated by the number of citations accrued by the development studies. The MODe has been used by researchers as a stand-alone assessment method to increase understanding of team behaviors in MTB meetings^11^–^13^ and also as a method alongside other performance metrics in complex interventional studies to assess the impact of interventions on aspects of team decision making.^10^ This flexibility has undoubtedly increased the uptake of the tool.

Use of the MODe,^7^ has come largely from dedicated academics with a specific interest in improving MTB processes. Since its development, the UK has had a move away from the implementation of top-down improvement in MTB transformation toward the adoption of solutions geared more to local challenges.^38^,^39^,^40^,^43^ A need therefore exists to equip health care professional to understand their own MTBs, and to identify solutions that work for them in their unique setting. Findings have clearly shown that the MODe requires a certain level of training before ratings can be reliably undertaken.^7^ It has become clear from our own experience of training health care professionals to use MTB improvement tools that such tools must have a short learning curve and be capable of quick administration in real-life MTB meetings.

The current study demonstrated that adequate reliability scores can be achieved by novice raters during a shorter period, offering improvements in feasibility. This suggests that MODe-Lite may offer health care professionals a simpler tool with a shorter learning curve that maintains the validity of the original tool. Further research is needed, however, for direct comparison of the learning curve and workload between MODe and MODe-LITE.

The ability of health care professionals at the grass roots to take ownership of improving the services they provide for patients is of growing importance. In the UK, MTBs have been urged to change the way they work in order to save a rapidly overburdened service.^38^,^43^ Guidance has been issued recommending that teams use evidence-based tools to understand and improve the way they work in order to meet local need.^38^,^43^ In the United States (and elsewhere), in which health care policy is less top-down, MODe-Lite offers a good starting point for an attempt to figure out team-based cancer care and enable teams to take a scientific approach to MTB development.

**Fig. 2.**
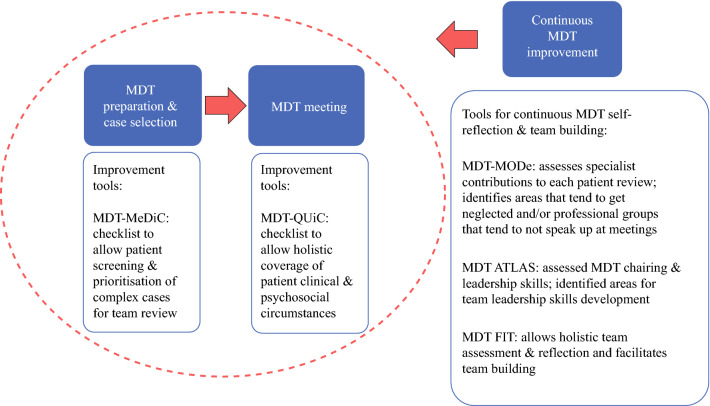
Schematic representing the phases of the multidisciplinary team working with application of quality-improvement tools. Reprinted with permission from^43^

As Fig. 2 suggests, MODe-Lite also could be used in a variety of ways, from stand-alone assessment of current working practices to a method alongside a more comprehensive tool kit.^12^,^20^,^41^–^43^ Either way, we recommend that potential assessors undertake training in the use of the tool that involves (1) learning about the tool, the scoring system, behavioral anchors, and how to mitigate biases given that this is an observational tool, (2) practicing scoring on real cases, either in a video format or in person (or both if available) and assessing inter-rater reliability in the process, and (3) scoring the cases for data collection purposes again using either a video or an in-person format once proficiency is reached (as assessed by adequate inter-rater reliability). Therefore, we recommend that users of MODe-Lite do the same as for the original MODe and hypothesize, given our findings, that the period of learning will be shorter than for the original tool.

An organization wishing to start using MODe-Lite might use video recordings for training and assessing interrater reliability if they are available. If videos are not available, the novice assessor can practice in real meetings. Either way, once a high level of interrater reliability is reached (>0.70),^26^ the assessor can begin to use the tool in real meetings for data collection and evaluation.

Streamlining of MTB processes, and MTB meetings specifically, is intended to allow more time for discussion of complex cases that truly benefit from a multidisciplinary approach. The MODE-Lite tool presents two potential opportunities for professionals seeking to undertake MTB improvement using evidence-based tools. First, concentration on those cases that benefit from a multidisciplinary approach endorses this way of working, and therefore places a requirement on MTBs to ensure that they are functioning as well as possible. Complex cases, by their nature, often have comorbidities or psychological or social challenges, as evidenced by the MeDiC tool with its stakeholder-driven development. 20 Decision-making in MTB meetings has consistently been found to underuse this type of information, to the detriment of patients.^1^,^5^ Similarly, MTB meetings are generally dominated by a small number of team members, with exclusion of others, particularly specialist cancer nurses.^1^,^9^ Nurses have a pivotal role in the care of cancer patients, and their input into the decision-making process in MTB meetings is critical to ensure the highest standard of care.^1^ In practical terms, deficiencies in information-sharing or under-representation of particular specialities at the MTB would show up as suboptimal scores for particular categories. Therefore, MODe-Lite might allow MTBs to quickly gain an understanding of the strengths and weaknesses of their MTB meeting processes, and how they can be improved.

Second, streamlining is intended to reduce the time and manpower required for MTB meetings. In addition, regular auditing and assessment using evidence-based tools is stipulated in guidance in the UK^38^ Streamlining therefore becomes both a driver and an opportunity to use tools to investigate and improve the way they work. For professionals with an interest in quality improvements, MODe-Lite provides a potential solution that gives a feasible, yet robust means of self-assessment.

Digitization of quality improvement tools, together with their integration into electronic medical record systems, will be important going forward. This will further improve feasibility and also facilitate aggregation of data over time across different tumor types or between MTBs. This will allow teams to better appreciate patterns of practice over time or space, perhaps in response to interventions designed to improve performance. It also may help comparison between different MTBs, facilitating bench-marking or accreditation.

## Study Limitations

Our findings need to be interpreted within certain limitations. First is the Hawthorne effect. In line with the ethical and regulatory approvals of participating NHS organizations in the UK, informed consent from team members was sought, which meant that they knew they were going to be filmed (i.e., there was no deception). To counteract this, a long-term approach to filming was adopted. Each team was filmed for 3 months. The first two meetings of each team were excluded from the analysis. The filming was performed discretely using a small GoPro camera, and the evaluators all were trained in use of the tools, which they scored in pairs blinded to one another’s observations.

Second, although tumor boards occur and are mandated for accreditation across various countries (e.g., American College of Surgeons Commission on Cancer), this study was conducted entirely in the United Kingdom. In large part, the domains identified through this rigorous study are fundamental to high-quality cancer care regardless where the care is delivered, although further validation in other cancer care systems is required. Finally, this study represents the most common cancers within the English NHS. Replication of the study in other cancers, teams, and health care systems is needed to support further generalizability of the findings.

## Conclusions

The MODe-Lite is a scientifically developed and validated tool for use by health care professionals to assess and improve MTB meetings. The learning curve appears to be shorter than for the previous version, with maintenance of its robust psychometric properties. It can be used alone or in conjunction with other quality improvement interventions to improve the care of cancer patients. Further work is needed to digitalize MODe-Lite and other quality improvement tools.

## Supplementary Information

Below is the link to the electronic supplementary material.

Supplementary file1 (DOCX 75 kb)
